# Gamma oscillations in visual statistical learning correlate with individual behavioral differences

**DOI:** 10.3389/fnbeh.2023.1285773

**Published:** 2023-11-08

**Authors:** Szabolcs Sáringer, Ágnes Fehér, Gyula Sáry, Péter Kaposvári

**Affiliations:** Department of Physiology, Albert Szent-Gyögyi Medical School, University of Szeged, Szeged, Hungary

**Keywords:** statistical learning, EEG, gamma band, implicit learning, behavior

## Abstract

Statistical learning is assumed to be a fundamentally general sensory process across modalities, age, other cognitive functions, and even species. Despite this general role, behavioral testing on regularity acquisition shows great variance among individuals. The current study aimed to find neural correlates of visual statistical learning showing a correlation with behavioral results. Based on a pilot study, we conducted an EEG study where participants were exposed to associated stimulus pairs; the acquisition was tested through a familiarity test. We identified an oscillation in the gamma range (40–70 Hz, 0.5–0.75 s post-stimulus), which showed a positive correlation with the behavioral results. This change in activity was located in a left frontoparietal cluster. Based on its latency and location, this difference was identified as a late gamma activity, a correlate of model-based learning. Such learning is a summary of several top-down mechanisms that modulate the recollection of statistical relationships such as the capacity of working memory or attention. These results suggest that, during acquisition, individual behavioral variance is influenced by dominant learning processes which affect the recall of previously gained information.

## 1. Introduction

Our sensory system is constantly bombarded with a great amount of environmental information. Some of this information is not random; relationships can be discovered between them. Statistical learning (SL) is the ability to extract the relationships underlying environmental stimuli without reward and build them into our internal representation of the environment, which later can be used to process incoming information more effectively. Although the phenomenon has been known for decades, the term “statistical learning” was coined in the domain of language learning (Saffran et al., 1996). Since then, the definition has been broadened as SL has been demonstrated in linguistic (Pinto et al., 2022) as well as in non-linguistic paradigms (Henin et al., 2021) using several stimulus modalities, including visual (Fiser and Aslin, 2002; Kaposvari et al., 2018), auditory (Saffran et al., 1999), tactile (Conway and Christiansen, 2005), and even multimodal designs (Seitz et al., 2007).

Aside from modalities, SL has been demonstrated across stimulus complexity and task complexity, age (Nemeth et al., 2013; Bertels et al., 2015; Zwart et al., 2019), IQ (Gebauer and Mackintosh, 2007; Kaufman et al., 2010), and even species (Santolin and Saffran, 2018). This universality supports that SL is a general neural process and possibly fundamental in the creation of internal representations of the environment (Armstrong et al., 2017). Despite the crucial role of SL in sensory processing, many studies have reported mixed behavioral results, where participants’ performance varied greatly, ranging from outstanding to poor results (Turk-Browne et al., 2010; Franco et al., 2015; Batterink and Paller, 2017; Juhasz et al., 2019). The underlying reasons for such differences in individual affinity towards statistical relationships in an elemental processing mechanism remain unknown. Although numerous studies have aimed to find a link between SL and factors such as age, intelligence, and certain cognitive functions, such as attention (Conway, 2020), working memory (Janacsek and Nemeth, 2013; Lengyel et al., 2019, 2021), or decision-making strategies (Harlé et al., 2015), only a few studies have aimed to find the neurological traces of interpersonal affinity differences in SL.

An often-used paradigm exposes subjects to an auditory sequence where tones create triplets that always occur together in the sequence. Using this paradigm, a difference can be discovered in the N100 and N400 components of EEG data. However, the appearance of this change is not consistent over time, since participants with good behavioral results showed it earliest while this difference was not detectable in the group with the lowest behavioral performance (Abla and Okanoya, 2009). In an EEG study using frequency tagging, a positive correlation was found between the normalized power of a left anterior and a right occipital cluster (Buiatti et al., 2009). Moreover, there was a positive correlation between the behavioral results and the intertrial phase coherence (ITPC) of the left pre-central gyrus and the right temporo-frontal area. Individual behavioral differences were explained as the previously acquired information emerged as explicit knowledge on different levels (Moser et al., 2021). Beta activity has been correlated with the behavioral results of SL; a 19–21 Hz oscillatory activity difference was found between within-pattern and between-pattern transitions. This difference emerged before stimulus presentation and the pre-stimulus beta-power increased during triplet transitions (Bogaerts et al., 2020).

In the current study, we aimed to find neural responses that correlate with the performance of the participants in SL task. The variance of the function that is related with the neural response can explain the interindividual differences. We used a previously published visual SL paradigm (Sáringer et al., 2022) and adapted it to EEG to find the cortical changes associated with SL, particularly the oscillatory patterns correlating with the individual results of an offline familiarity test. A substantial part of the suspected processes that might be responsible for inter-individual differences belongs to executive functions. These functions are typically linked to the frontoparietal region, and in the time-frequency spectrum, the related signal response emerges with late latency in the high-frequency range (Reber et al., 2003; Herrmann et al., 2004; Smittenaar et al., 2013). We identified a high-frequency oscillatory activity that correlated with the participants’ behavioral results and could be a potential marker of their affinity towards environmental regularities. Afterward, we traced the scalp distribution of said changes. Based on this activity we theorize underlying top-down mechanisms influencing interpersonal differences in SL.

## 2. Materials and methods

### 2.1. Participants

Seventeen (9 females, mean age: 25.7 y) and thirty (16 females, mean age: 26.4 y) volunteers participated in the pilot behavioral study and in the EEG study for course credits, respectively. Based on the results of the pilot study (A′ (mean ± SD) = 0.6 ± 0.17, Cohen’s d = 0.59) *a priori* calculation (*ɑ* = 0.05, *β* = 0.95) showed the need for a sample size of 33. All participants provided written, informed consent; all stated having a correct or corrected-to-normal vision and no history of epilepsy or other neurological diseases. One participant was excluded due to extremely high noise in the recording. The study protocol was approved by the Human Investigation Review Board of the University of (266/2017-SZTE).

### 2.2. Sequence design

**Figure 1:**
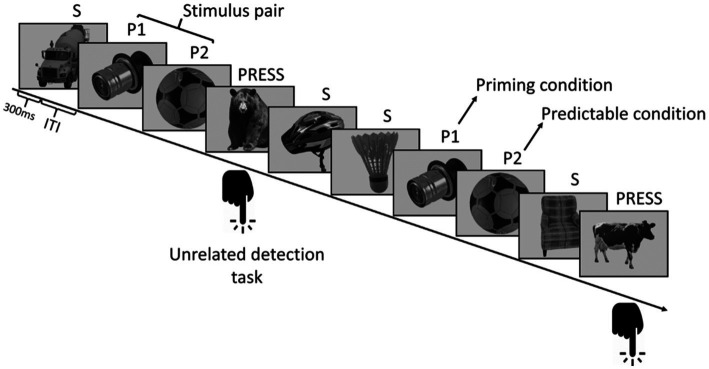
Stimulus sequence structure. Eight out of the 16 pictures of everyday objects formed four stimulus pairs; their members always followed each other with a transitional probability of one. The remaining eight stimuli were part of the control, S conditions, which had no statistical relationship with any other picture. Pictures were displayed for 0.3 s; a jittered intertrial interval (ITI) was used (0.7–1.2 s).

We adapted a previously described paradigm (Sáringer et al., 2022). In each run, 412 trials were presented to the participants: 16 images of objects were presented 25 times, and 12 pictures of animals were randomly inserted into the stream; these 12 pictures were target stimuli. A run was divided into two parts. During the first part, all 16 objects were presented 10 times randomly under the constraint that a stimulus could not appear again until after three other stimuli had been presented. The second part had 15 cycles; each cycle contained all 16 stimuli. In this part, regularities were hidden in the sequence. Eight pictures formed four stimulus pairs, always following each other in a fixed order. The remaining eight stimuli served as control; they had no statistical relationship with any other stimulus above chance. Thus, there were three conditions: Condition P1, P2, and S. Condition P1 refers to the first member of the stimulus pair which always precedes the predictable stimuli. Condition P2 refers to the second member of the stimulus pair which becomes predictable. Condition S indicates the remaining eight control stimuli (Figure 1).

### 2.3. Task

**Figure 2:**
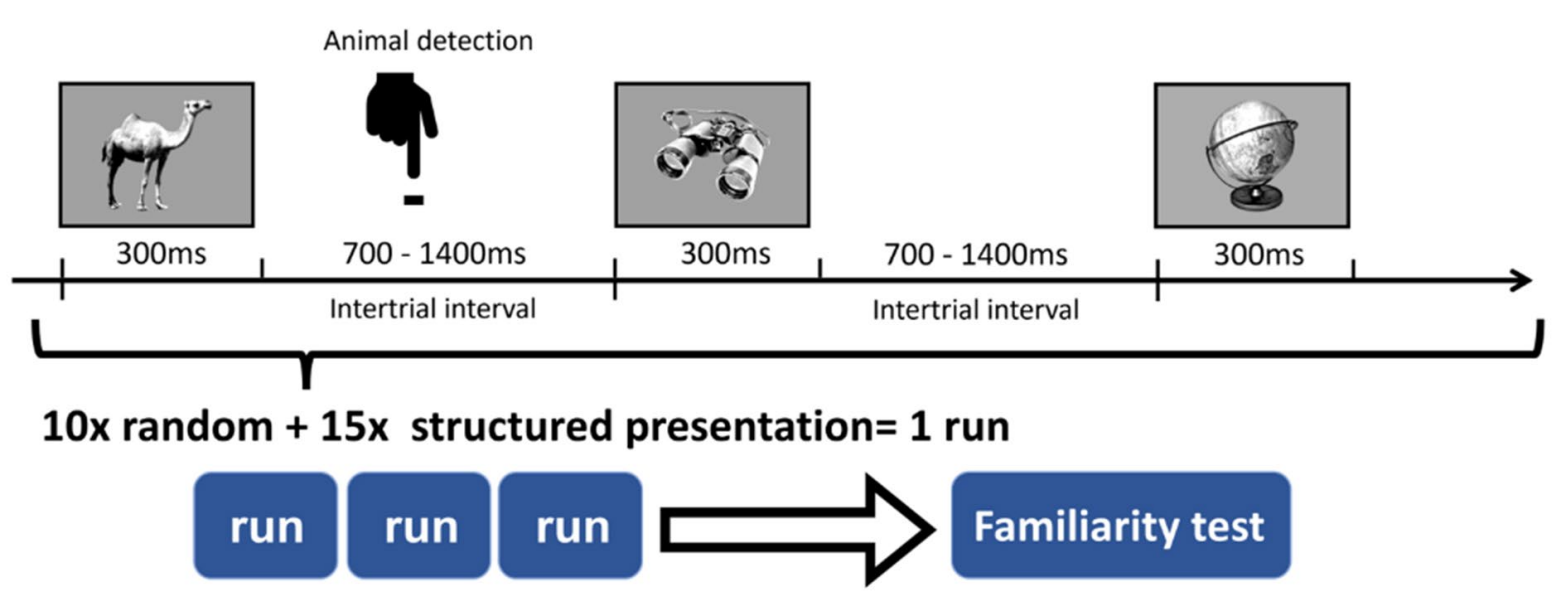
Detection task during the sequence. Participants were asked to focus on the images and indicate the appearance of an animal with a button press.

In each run, 12 images of animals were randomly inserted into the sequence. Participants were informed that they were going to view a stream of images representing everyday objects and animals. Their task was to indicate the appearance of an animal with a button press as soon as they could (Figure 2). This task aimed to focus the subjects’ attention on the visual stream, and at the same time, it helped to keep the regulatory information implicit.

### 2.4. Familiarity test

After the sequence presentation, participants performed an offline familiarity test to evaluate their gained knowledge about the associated stimuli pairs in a two-alternative forced choice (2AFC) design. The test consisted of four presentations of eight stimulus pairs, 32 presentations in total. Four pairs were previously seen and the rest were newly added by switching one member of the original pairs from the same position. The members of each pair were presented in the same way as during the original sequence. After presenting both members, participants had to indicate whether the presented stimulus pair was familiar or not with a keyboard button press (‘S’: not familiar, ‘K’: familiar). The next pair was shown after the subject had given an answer.

### 2.5. Stimulus presentation

Gray-scale images of everyday objects were presented to the participants in a stream. Sixteen images of objects and 12 of animals were selected from the Bank of Standardized Stimuli (BOSS) (Brodeur et al., 2010, 2014) for each run. All stimuli were displayed at approximately 7.5° × 7.5° in visual angle. Stimuli were presented in Psychtoolbox, MATLAB (Brainard, 1997) on an ASUS ROG Swift PG248Q Monitor (24″, FHD, 1920 × 1,080). Participants were seated approx. 50 cm from the screen. Pictures were displayed for 0.3 s and a jittered intertrial interval was used (0.7–1.2 s).

### 2.6. Procedure

First, we run a pilot behavioral experiment to determine whether our paradigm works and SL is detectable with a familiarity test. Note that the original paradigm (Sáringer et al., 2022) was able to measure SL online because the subjects needed to indicate the category of each presented stimulus by a button press. Here, we modified the paradigm to exclude the motor component in the EEG signal during the training period. At the beginning of this pilot study, participants were instructed that they should press number 1 on the numeric keyboard when seeing an animal among a stream of pictures. After one run (412 trials in total, approximately 7 min long), they performed the familiarity test, where they were informed about the hidden regularities.

After the pilot behavioral study, we performed the EEG study during which stimuli presentation was coupled with EEG recording. To accumulate data and reduce variance, we performed three runs (1,236 trials, ~25 min). For all subjects in each run, a newly generated sequence with new regularities (pairs) was used. The total presentation of every picture was 25 times (10 times in the random sequence and 15 times in the structured sequence), thus the pairs were presented 15 times accordingly. Between runs, participants could take a few minutes break to relax and reduce fatigue. At the end of the three runs, participants were informed about the regularity and performed a familiarity test based only on the regularities seen in the last run.

### 2.7. EEG data acquisition

EEG data were recorded using a 64-channel Biosemi Active II system with a sampling rate of 2048 Hz. An electrooculogram (EOG) was recorded using four channels 1 cm above and below the left eye and the outer canthus of both eyes.

The preprocessing pipeline was implemented in EEGLAB (Delorme and Makeig, 2004). First, based on visual inspection, channels with compelling artifacts and noise were interpolated. Then, a 1–80 Hz bandpass filter and a 48–52 Hz Notch filter were applied. The data were re-referenced to the grand average and resampled to 200 Hz. Eye movements and other artifacts were excluded using EyeCatch software (Bigdely-Shamlo et al., 2013) and Multiple Artifact Rejection Algorithm (MARA, Winkler et al., 2011, 2014). We removed the EOG channels from the clean data and epochs were defined: from 0.7 s before to 1.7 s after the beginning of stimulus presentation.

Clean and segmented data were further processed using Fieldtrip (Oostenveld et al., 2010). Event-related potentials (ERP) were baselined to 0.2 s before stimulus presentation and inspected between −0.2 and 1 s. ERP was calculated for each subject, condition, and channel and used for further analysis.

A time-frequency analysis of the data was performed via Morlet wavelet analysis in the time-frequency window of −0.7 to 1.7 s and 2–80 Hz. Hanning taper and padding were applied. For the inspection of lower frequencies (8–30 Hz) range cycle number of 4 and for higher frequencies (30–80 Hz) range cycle number of 10 was used. The power was baselined to 0.4–0.2 s before stimulus presentation and given in dB. Then the spectral data were averaged across trials.

ITPC was calculated for the same time-frequency window as in the time-frequency analysis. Frequency decomposition was performed via Morlet wavelet analysis with a cycle number of 10 (high-frequency range). The ITPC value can range between 0 and 1, where 0 means high variance in phase angles and 1 indicates that all trials have the same phase angles.

### 2.8. Statistical analysis

On the familiarity test data, the modified Grier’s formula was applied (Eqs 1, 2) (Grier, 1971, Aaronson and Watts, 1987), where HIT means the hit probability of the subject and FA signifies the false alarm probability of the subject. This formula describes the subjects’ sensitivity in a 2AFC task as a value A’, a variable between 0 and 1. Chance performance is 0.5 A’, approaching 1 indicates improved performance in a 2AFC test. Equation 2 is used when a participant’s hit probability is lower than their false alarm probability. We calculated the A′ value of every subject and used a one-sided *t*-test to evaluate whether the population mean is significantly different from 0.5. In addition, we divided the participants into two groups based on the result of the familiarity test. EEG volunteers, who performed >0.5 on the test were labeled AC for above-chance performers; the remaining subjects were labeled C for chance performers.


(1) $$A^{\prime }=\frac{1}{2}+\frac{\left(\mathrm{HIT}-FA\right)\times \left(1+\mathrm{HIT}-FA\right)}{4\mathrm{HIT}\times \left(1-FA\right)}$$

(2) $$A^{\prime }=\frac{1}{2}+\frac{\left(FA-\mathrm{HIT}\right)\times \left(1+FA-\mathrm{HIT}\right)}{4FA\times \left(1-\mathrm{HIT}\right)}$$

For ERP comparisons, the Fieldtrip toolbox was used. Permutation statistics with cluster-based correction were applied where data points within the cluster were connected through time and channels. The result of dependent t-tests determined the cluster size, where the significance threshold was set to 0.0167 (one-third of 0.05, after Bonferroni correction for the multiple comparisons among the three conditions). After 1,000 permutations, the 95th percentile of the summed t values within a cluster were accepted as significant.

For the time-frequency data analysis, a window of interest was chosen in the mean spectral data of all subjects, channels, and conditions following the method of Bogaerts et al. (2020). To confirm that the power in this window can contribute to the variance in the performance of the offline test, the participants’ behavioral results were correlated with the average power of our window of interest using Pearson’s correlation.

To confirm the results of the correlation and to specify the origin of the increased oscillatory activation, permutation-based statistics were applied to the time-frequency data with Bonferroni and cluster-based correction as described above. The clusters were determined across time, frequency, and channel dimensions. For the comparisons, the two groups [above chance (AC, *n* = 14) and chance (C, *n* = 15) performers] were contrasted. Then, the three conditions (P1, P2, and S) were contrasted within the AC group.

To test the phase-lock to the stimulus onset time, after averaging the ITPC values in the observed time-frequency window and same size window in the pre-stimulus baseline period, they were compared with Wilcoxon signed-rank test where ɑ was set to 0.05.

## 3. Results

### 3.1. Behavioral pilot study

**Figure 3:**
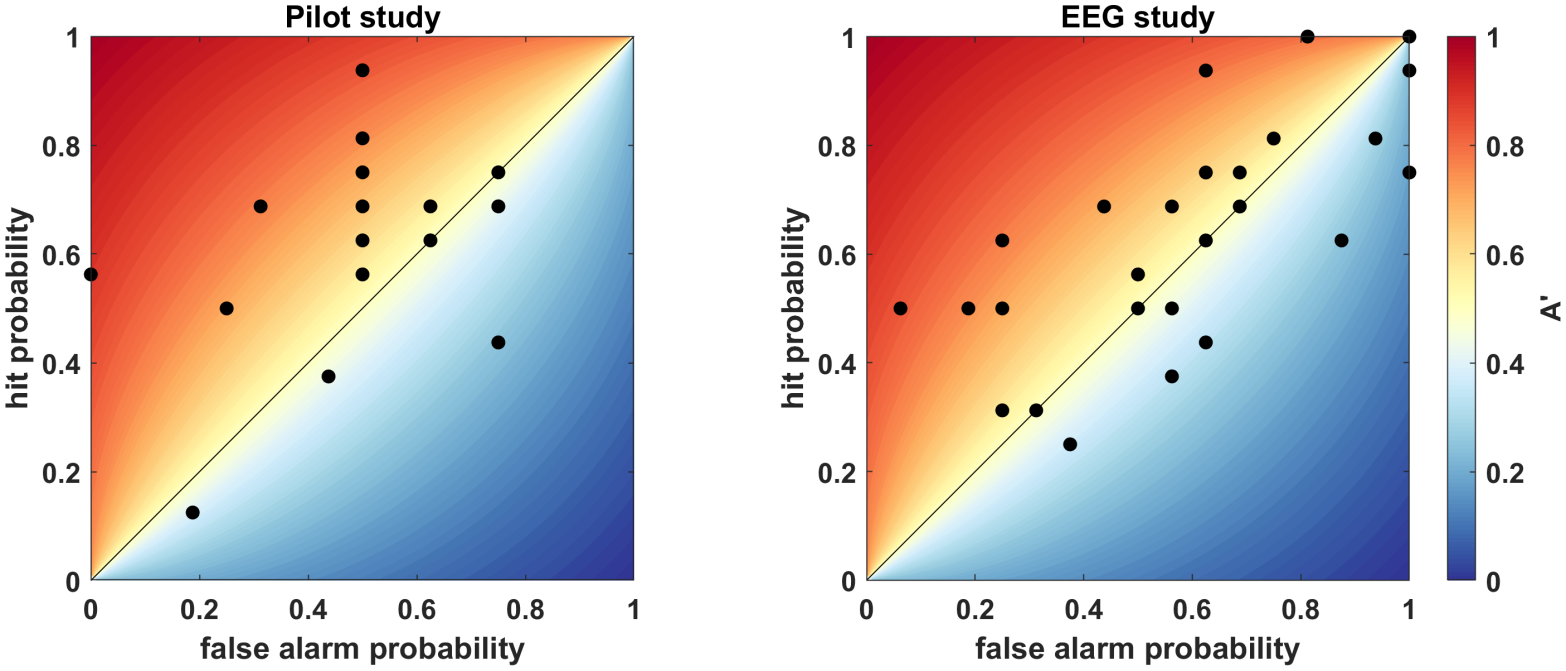
Individual A′ values indicating accuracy, where each dot represents the result obtained by a single participant. The hit probability (true pair/“familiar” answer) is displayed against the false alarm probability (false pair/“familiar” answer). The color bar indicates the A′ value from 0 to 1, while the diagonal line is the 0.5 chance performance. Left: behavioral pilot study. Right: behavioral results of the EEG study.

To assess the adequacy of the above-described paradigm, we conducted a pilot behavioral study on 17 participants. The sample mean of A’ was significantly higher than 0.5 (A′ (mean ± SD) = 0.6 ± 0.17, Cohen’s d = 0.59, *t* (16) = 2.44, *p* = 0.0132; Figure 3). Accordingly, we used this paradigm for the subsequent EEG study.

### 3.2. Behavioral results of the EEG study

In the EEG study the sample mean was >0.5 (A′ (mean ± SD) = 0.53 ± 0.18, Cohen’s d = 0.18; Figure 3), but not significantly so (*t* (28) = 1.002, *p* = 0.1623). Out of the 29 people, one person explicitly reported that she did not comprehend the task and additionally we speculate three people misunderstood the task since they answered only “not familiar.” Still, these subjects were kept in the analysis, so we do not increase bias in the study. Although the analysis of the behavioral results was not significant, it showed the same tendency as the pilot study, where we could confirm the efficiency of the paradigm. Hence, we pursued the analysis of the EEG data with 29 participants. According to the outcome of the familiarity test, the participants were divided into two groups: AC, above chance performers, who achieved >0.5, and C, chance performers, who resulted in ≤0.5 on the familiarity test.

### 3.3. Analysis of the event-related potentials

No significant clusters were found in the data of all subjects when contrasting the post-stimulus intervals of the three conditions. Neither the analysis of all data of the AC group nor that of the data from the last 10 repetitions (6 to 15) in the same group showed significant clusters.

### 3.4. Spectral analysis

**Figure 4:**
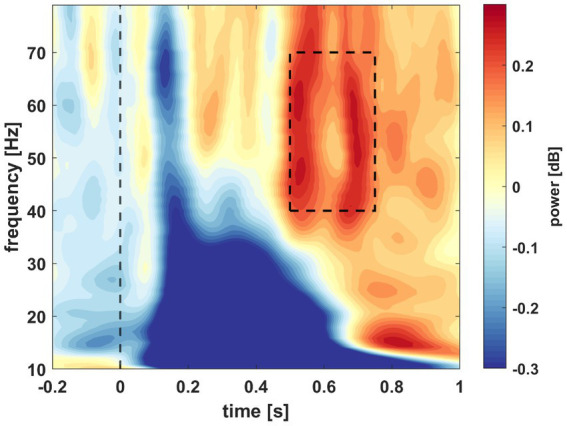
Spectral power averaged across all subjects, conditions, and channels. Power is illustrated by time in s (x-axis) and frequency in Hz (y-axis) in the window of 0.2 s before stimulus presentation and 1 s post presentation and from 10 to 80 Hz. The dashed window indicates the selected window of interest.

**Figure 5:**
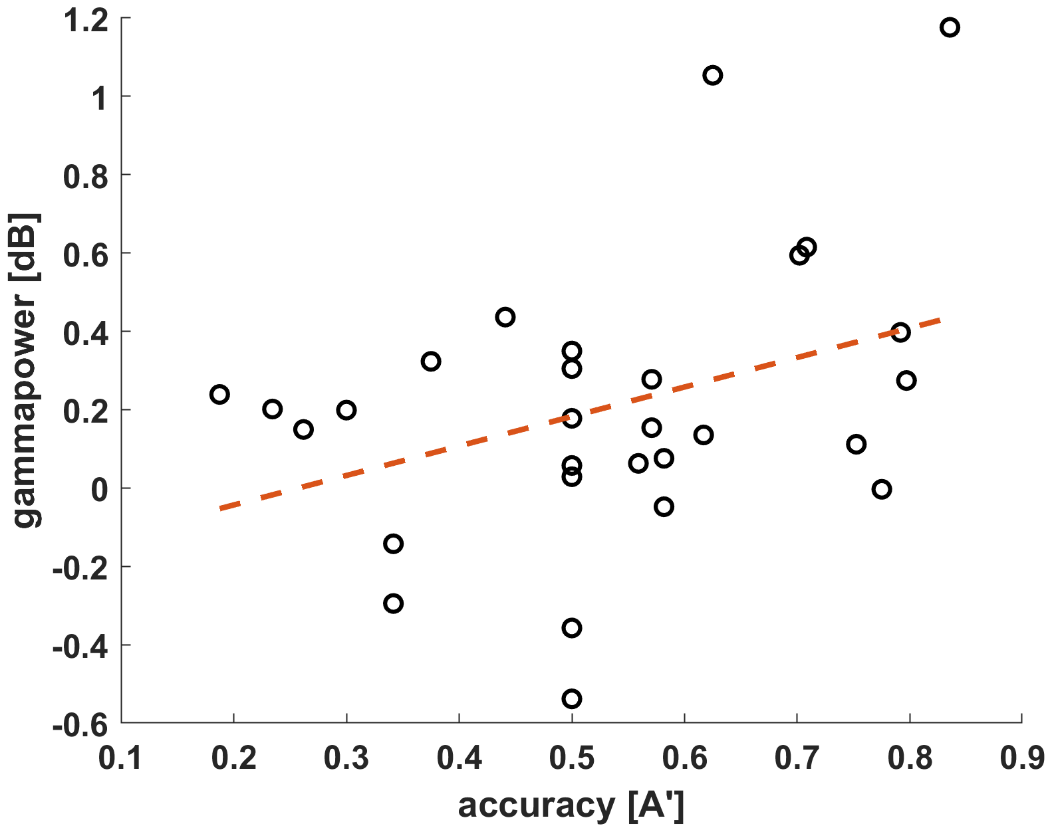
Correlations of the A′ value and average gamma power (40–70 Hz, 0.5–0.75 s window, all channels) of all participants (*n* = 29, *r* = 0.3705, *p* = 0.0478). Gamma power in dB (y-axis) is displayed by the participants’ individual A′ values (x-axis).

To determine a window of interest we followed the methodology of Bogaerts et al. (2020). The time-frequency data was averaged over all subjects, conditions, and channels. A window of interest was selected in the gamma range (40–70 Hz) from 0.5 to 0.75 s after stimulus presentation (Figure 4). Then, we investigated the relationship between the individual gamma powers (averaged across all conditions and channels) and the behavioral results tests of the participants. The average powers of the window of interest were assigned next to the A’ values of each participant. Pearson’s correlation coefficient was calculated based on the data distribution. The test yielded significant results with a coefficient of *r* = 0.3705 (*n* = 29, *p* = 0.0478; Figure 5). As mentioned previously, the behavioral data of four participants do not reflect their actual acquisition of the associated pairs due to misconception. In a reanalysis of the correlation, we excluded these participants to verify the observed relationship between the behavioral and electrophysiological data without this skewing factor. In this case, with only 25 subjects, the correlation statistics still yielded significant results (*n* = 25, *r* = 0.4494, *p* = 0.0242). Based on this, we decided to keep the electrophysiological data of these four participants and continued the EEG analysis with 29 subjects.

**Figure 6:**
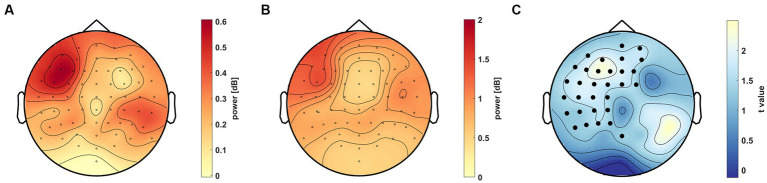
Scalp distribution of the 40–70 Hz gamma activity in the 0.5–0.75 s time window. **(A)** Power difference (colorbar) between the AC and C groups on the scalp. **(B)** standard deviation of the difference, where $\sigma_{\mathrm{diff}}=\sqrt{\sigma_{1}^{2}+\sigma_{2}^{2}}$. **(C)** Difference illustrated as t values (colorbar) on the scalp. Channels in the significant cluster (*t*_sum_ = 860.57, *p* = 0.041) are marked as black dots (*p* < 0.05).

In order to establish the spatial distribution of the 40–70 Hz gamma activity, we compared the AC and C groups. The average power in all conditions was used to perform permutation-based statistics across the scalp in the previously described 0.5–0.75 s period. After correcting for multiple comparisons, a significant cluster emerged in the left frontoparietal region (*t*_sum_ = 860.57, *p* = 0.041, Figure 6). According to the analysis, the AC group (mean ± SEM = 0.35 ± 0.07 dB) has a higher average gamma power than the C group (mean ± SEM = 0.08 ± 0.05 dB).

**Figure 7:**
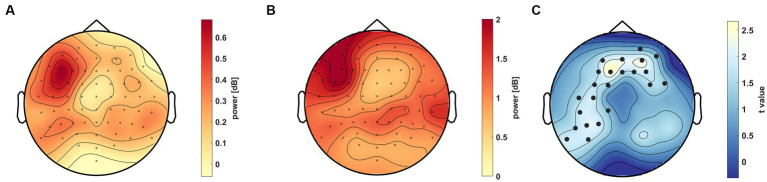
Scalp distribution of the 40–70 Hz gamma activity in the 0.5–0.75 s time window between Condition P1 and S within the AC group. **(A)** Power difference (colorbar) of the two conditions across the scalp. **(B)** standard deviation of the difference, where $\sigma_{\mathrm{diff}}=\sqrt{\sigma_{1}^{2}+\sigma_{2}^{2}}$. **(C)** Difference displayed as t values (colorbar), where significant channels (t_sum_ = 681.30, *p* = 0.021) are marked as black dots (*p* < 0.0167, after Bonferroni correction).

We compared oscillations in the same range (40–70 Hz) and in the same time window (0.5–0.75 s after stimulus presentation) on the scalp to determine whether this high-frequency activity is constant across all conditions or condition-specific. No significant cluster appeared including all subjects in the analysis. Nonetheless, we performed the analysis separately on the AC and C groups. Although no significant cluster could be discovered within the C group, a difference was detectable between condition P1 and S in the AC group (*t*_sum_ = 681.30, *p* = 0.021, Figure 7). The distribution of gamma oscillations between conditions was remarkably similar to the previously described activity differences between the AC and C groups as it appeared in the left frontoparietal region.

**Figure 8:**
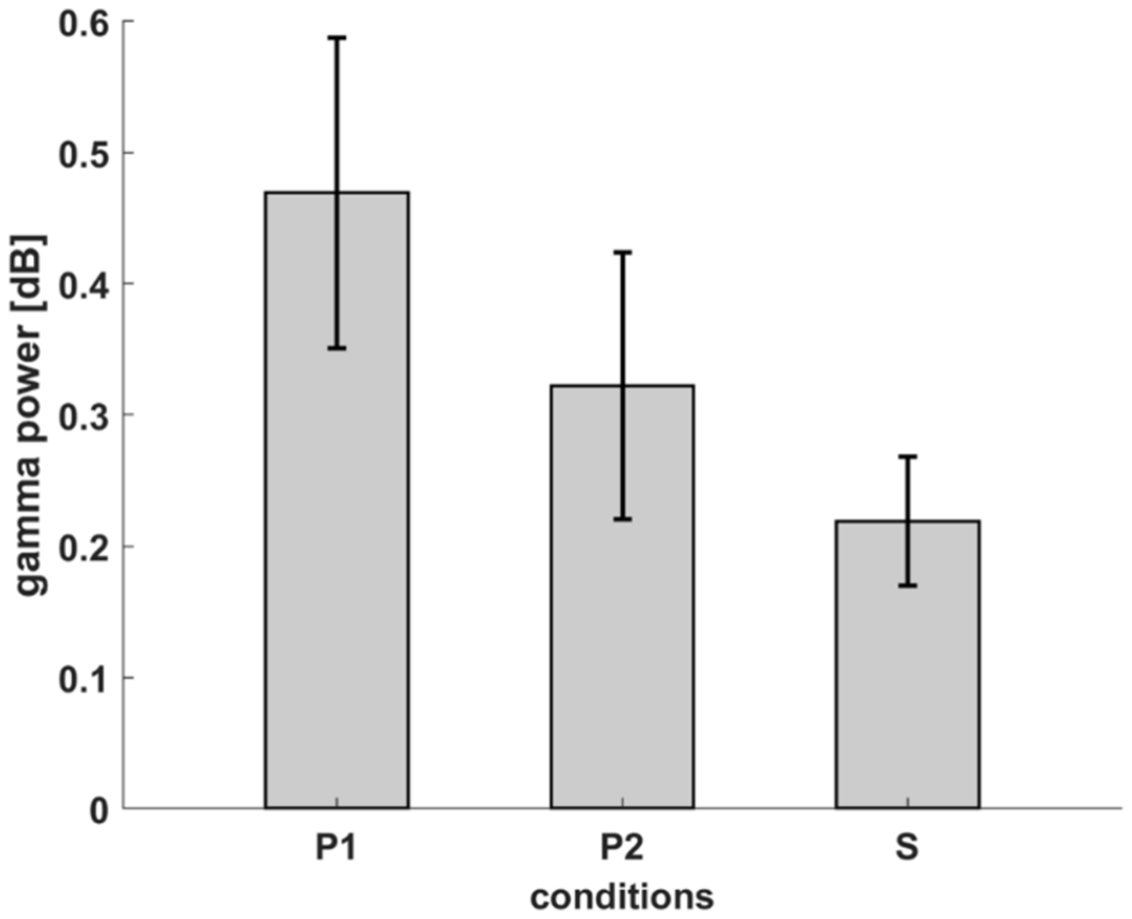
Average power in each condition within the time-frequency window of interest (0.5–0.75 s, 40–70 Hz). The error bars indicate SEM.

The finding that condition P2 was not significantly different from either condition was not in line with our expectations. If there is no change during the processing of P2 stimuli, condition P2 should have had similar dynamics as condition S, being significantly different from condition P1. If the underlying process does change due to SL, Condition P2 should have been significantly different from condition S. To understand why condition P2 does not differ from the other conditions, we observed the average gamma power in the window of interest (40–70 Hz,0.5–0.75 s post-stimulus) in each condition. The mean gamma power was greatest in Condition P1 and lowest in condition S [P1 (mean ± SEM) = 0.4691 ± 0.1184 dB, S (mean ± SEM) = 0.219 ± 0.0492 dB]. In condition P2, the gamma power (P2 (mean ± SEM) = 0.3222 ± 0.1015 dB) lies in between, which can explain why we could not detect significant differences between P1 and P2 or P2 and S in the cluster analysis (Figure 8).

The ITPC of the time-frequency window of interest (0.5–0.75 s, 40–70 Hz) in group AC (Median: 0.0698 IQR: 0.0052) in condition P1 was compared to the ITPC of the pre-stimulus baseline window (40–70 Hz, −0.4 to −0.15 s) in the same group and condition (Median: 0.0683, IQR: 0.0048). The difference in median coherence was not significant (*N* = 14, *z* = −0.2824, *z*/√*N* = −0.075, *p* = 0.78).

After establishing the results found in the gamma range, we tracked differences in the theta (4–7 Hz), alpha (8–12 Hz), and beta (13–30 Hz) ranges using the same time window. These frequency bands showed no significant or even tendentious difference between groups or between conditions. Based on these findings we did not pursue any analysis beyond the previously settled gamma window.

## 4. Discussion

Our aim was to find electrophysiological markers that correlate with the accuracy in a VSL paradigm and can contribute to clarifying the great interpersonal differences in performance in SL studies. We assessed an unsupervised VSL paradigm with determined regularities (associated stimuli pairs) in a temporal sequence. We found that gamma activity correlated with performance in the familiarity task (Figure 5). This activity in the low gamma range (40–70 Hz) over the left frontoparietal region increased in the AC group between 0.5 and 0.75 s post-stimulus (Figure 6). Furthermore, this gamma oscillation was higher in condition P1 compared to the control condition and not phase-locked to the stimulus onset time (Figure 7). Considering the predictive role of Condition P1, the latency of the gamma activity relative to the stimuli, and its correlation to performance, this induced gamma activity appears related to SL; however, the function of this high oscillatory activity still needs to be clarified.

### 4.1. Behavioral findings

In line with previous research, great variance was observed in participants’ performance (Turk-Browne et al., 2010; Franco et al., 2015; Batterink and Paller, 2017; Bogaerts et al., 2022). High variance exists both within individual studies and between different studies despite the fact that most studies, including ours, measured a relatively homogeneous population of university students between 20 and 30 years old. In the pilot behavioral study, we found a significantly different mean population performance from chance, which proved that the regularities were acquired, and our paradigm was suitable to detect SL. Similarly, to the pilot study, the mean population performance was higher than chance in the EEG study, however, behavioral results failed to reach significance (Figure 3). A possible reason for this result could be fatigue. Further possible factors resulting in null finding could be that contrarily to the single run of the pilot study, participants were exposed to three runs in the EEG study, which aside from the longer time of the task also meant three times more stimuli pairs to learn even if the successful acquisition was tested only in the last run. Notwithstanding, the lack of great effect size in the familiarity test does not influence the cortical activity related to the acquisition process of statistical relationships.

### 4.2. The role of gamma oscillations

To adequately interpret the role of the observed gamma power change we place our findings in literature. Post-stimulus gamma activity was defined as early and late gamma activity previously. Early gamma activity was defined as a transient evoked activity with phase-locked oscillation ending before 0.15 s after stimulus presentation. It is a correlate of basic perceptual processes based on the latency and the phase-locked nature of the oscillation (Pulvermüller et al., 1999). Late gamma activity is a longer, non-phase-locked oscillation, typically found between 0.2 and 1 s with great variance and in the low range between 30 and 80 Hz. In the *match and utilization model* for interpreting gamma oscillations, early gamma is linked to matching the incoming sensory information to past experiences while late gamma oscillation is linked to utilization (Herrmann et al., 2004). Utilization is described as a process during which, after matching, information “can be used for coordinating behavioral performance, for redirecting attention, or for storage in memory” (Herrmann et al., 2004). One aspect of utilization can be attention control: gamma activity has been linked to attention (Fell et al., 2003) and plays a role in top–down attentional control in the case of predictable stimuli (Gonzalez Andino et al., 2004). In addition, the role of attention in SL has been described as a reciprocal relationship, since attention to a regularity helps its acquisition, and attention can be guided to learned regularities through top-down mechanisms (Conway, 2020). Based on the frequency range (40–70 Hz), the latency (0.5–0.75 s), and also being a non-phase-locked activity, we interpret our findings as a late gamma activity involved in the utilization process.

Not only the latency and frequency range but the location of the oscillation helps to narrow down the spectrum of the suspected function. The earlier and more posterior the activity on the scalp the more likely it represents sensory functions, while later and more anterior activity is linked to executive functions (Reber et al., 2003; Smittenaar et al., 2013). However, the border between sensory and executive functions is not always clearly defined, particularly concerning SL. Likewise, the definition of utilization allows a variety of interpretations. Another theory, which defines model-free and model-based learning, draws a similar conclusion. Although overlapping with the match and utilization model, it links the processes to different brain regions. Model-free learning means stimulus-driven, incidental detection of environmental regularities in an unsupervised form without intention and is assumed to happen implicitly. The extraction of stimulus patterns is an essential element of SL that can be linked to bottom-up processes and appears in the posterior regions of the cortex. Meanwhile, model-based learning constructs an internal representation from the extracted regularities to execute goal-oriented behavior and modulate executive functions. It can be related to top-down mechanisms and appears in the anterior regions of the cortex (Daw et al., 2011).

The role of top-down mechanisms in anterior areas affecting SL was tested in a recent study (Ambrus et al., 2020), where the activity of the dorsolateral prefrontal cortex (DLPFC) was disrupted by transcranial magnetic stimulation during an alternating serial reaction time task. After 24 h, they found higher SL performance. The result is interpreted in a competitive framework due to an antagonistic relationship between model-based and model-free learning. DLPFC disruption decreased model-based learning, opening the door for model-free learning. Model-based learning is linked to the PFC (prefrontal cortex) and the hippocampus (Conway, 2020). The PFC is associated with the function of working memory and has the ability to keep the information available in a temporal design during a delay period between stimulus presentations. Thus, it seems essential in learning associated stimuli in time. The hippocampus has a possible role to build the information originating from incidental learning into the internal representational schemas (Cowan, 1988; Cowan, 2017).

Another study (Batterink et al., 2015) makes functional distinctions between brain areas and their results are in line with the previously mentioned model-free/model-based approach. Posterior areas were associated with bottom-up functions, meaning they have greater activity in case participants have to rely on the sensory processing of stimuli without pre-existing knowledge, while the anterior areas are more involved in top-down processes when participants can use previously acquired information about the upcoming stimuli. Their approach is very similar to the above-discussed model-free/model-based learning model, but they view these learnings as parallel processes, where their relationship is not necessarily competitive.

The familiarity test used in our paradigm is an offline, somewhat explicit measurement (Turk-Browne et al., 2005; Kim et al., 2009; Batterink et al., 2015). To achieve above-chance performance on the test, two requirements must be met. First, extraction of an observed environmental regularity and model building must be achieved. Next, the participant needs to recall explicit knowledge from this internal representation. In our view, the term utilization describes almost identical cortical processes as model-based learning. The temporal aspect of our results suggests that the observed gamma activity differences are correlates of the utilization process. The localization of the oscillatory activity supports the hypothesis that it is a correlate of model-based learning. In addition, these two terms are linked by their similar description, as well. Based on these observations we theorize that late gamma oscillation is the correlate of model-based learning.

However, we cannot rule out the possibility that the extraction of regularities and building of an internal representation happens in every participant since our paradigm cannot measure model-free learning processes. Independent of a possible competitive or parallel relationship between model-based and model-free learning processes, the gamma activity and its correlation to behavioral performance suggest that the level of model-based learning dominance can potentially explain interpersonal differences in offline tests. However, this conclusion does not fully address our original question, since model-based learning and utilization involve several parallel cortical and subcortical functions, such as attention and working memory. Thus, the precise cortical process and the origin of the gamma oscillation need to be confirmed in further studies.

## 5. Conclusion

In conclusion, we aimed to find cortical correlates of the behavioral performance of an offline familiarity test in a temporal VSL paradigm to clarify the ambiguous results obtained in frequently used offline tests. We found that the familiarity test results correlated with a frontoparietal post-stimulus low gamma activity. Based on the latency and frequency range of the oscillation (0.5–0.75 s, 40–70 Hz), we identified this activity as a late gamma oscillation. According to the literature, this late gamma activity appears to be a correlate of model-based learning, which is required for information recall during an offline test. Hence, we propose that the dominance level of model-based top–down processing contributes to the experienced performance variances.

## Data availability statement

The raw data supporting the conclusions of this article will be made available by the authors, without undue reservation.

## Ethics statement

The studies involving humans were approved by Human Investigation Review Board of the University of Szeged. The studies were conducted in accordance with the local legislation and institutional requirements. The participants provided their written informed consent to participate in this study. Written informed consent was obtained from the individual(s) for the publication of any potentially identifiable images or data included in this article.

## Author contributions

SS: Data curation, Methodology, Software, Writing – original draft, Formal analysis, Validation, Visualization. ÁF: Data curation, Writing – original draft. GS: Supervision, Writing – review & editing. PK: Supervision, Conceptualization, Data curation, Methodology, Software, Writing – original draft.
